# Indents

**Published:** 1913-08-15

**Authors:** 


### INDENTS.

☞Brief Articles of Technical or Scientific Value will receive notice and comment. Contributions invited.

*Intern Work Required for Graduation.* Rush Medical College will require all students entering for the summer quarter of 1914 and thereafter to register for a five-year medical course. The fifth year is to be spent by the student either in graduate work in one of the departments of the college, or as an intern in an approved hospital under the constant supervision of the college faculty. The fifth-year course was established in 1905, but was made optional owing to the wording of the Illinois practice act which required that only graduates of medical schools could serve as interns in hospitals. The practice act has since been amended, thereby permitting the college to require the intern year prior to granting the degree. This makes three medical colleges which have definitely decided to require the fifth year of intern or other clinical work. In 1910 the University of Minnesota adopted the requirement for all students entering in the fall of 1911 and thereafter. A few weeks ago also Leland Stanford Junior University Department of Medicine announced a similar requirement for all students entering in 1914 and thereafter. There is also one state, Pennsylvania, which after January 1, 1914, will require that every candidate in order to be eligible to receive a license to practice in that state must have first obtained an internship, which, however, need not necessarily have been obtained prior to graduation, but must have been taken in a hospital approved by the Pennsylvania board. There is much to say in favor of the college requiring the internship prior to granting the degree. The chief advantage is the constant check which the college will or should have on the intern's work; this will be in marked contrast to the haphazard manner in which interns, in some instances at least, carry out their obligations to the hospitals.

This new arrangement gives assurance of a better training for the intern, of more satisfactory intern service for the hospital, and of safer and more thorough care of the patients in the hospital. Altogether, this next great step in the advancement of medical education, which is apparently to be brought about in the near future, will provide for the public more thoroughly qualified and skilled practitioners of medicine.—*Journal A. M. A.*, June 14, 1913.

*Medical Research and Medical Education.* The announcement was made last week that a million dollars had been given to develop medical research at the University of California. This generous gift will provide a research institute on the Pacific Coast comparable with the Rockefeller Institute in New York City. It will differ from the eastern institution, however, in that it will be closely connected with a medical school. This is as it should be. While medical research has no place in the undergraduate medical curriculum, its presence in the medical school is of the highest importance. It creates an atmosphere of investigation with which every medical student should become imbued. It is carried on by a group of workers who are also medical teachers and the best teachers are always those who are also actively engaged in medical research. Medical research is, in fact, the very soul of medical education; without it the medical school is likely to become a mere machine, teaching the discouraging facts regarding morbid processes, sickness and death without the inspiration of encouragement and hopefulness which comes from delving after new truths and the discovery of new means of alleviating disease and pain. In the medical school without research the highest ideal held by the student may often be the financial returns he may obtain from his practice. In the research school, on the contrary, the student, by the time he graduates, is led to realize that his study of medicine has just begun and also to realize how boundless are the possibilities for usefulness in the field of medicine. It is undoubtedly the latter school which turns out the best practitioners of medicine. It also turns out those who are better prepared to serve their communities in promoting improved sanitary measures whereby needless sickness and suffering are prevented. Our congratulations are extended to the people of California and of the Pacific Coast on their recent good fortune.—*Journal A. M. A.*, June 14, 1913.

*Saddle Bridges.* A word of caution in regard to the indiscriminate use of the saddle is needed for that is by all means to be discouraged. It wants to be used cautiously and under proper indications, but when intelligently applied I believe it is the most sanitary bridge which we can make in the way of a fixed bridge. For years I have bitterly opposed the fixed bridge from a sanitary point of view in every type, but we are compelled in a great majority of cases to put in fixed bridges, but when we have to do so, we must do the best we can with reference to the various requirements. With reference to the saddle, I spoke to Dr. Goslee to-day and asked him if I could quote him as favoring the saddle, and if he favored these individual saddles, what he would say in opposition to the use of them, he replied, "Roach, the man who opposes the individual saddle is the man who has not had a very extensive experience with it." I believe that is true, and I quote Goslee because I know of his large experience. He is using these saddles in connection with his tooth.

Dr. MacBoyle criticises the fixed saddle on the ground that he had never seen one under which there was not either a congested inflammed or at least an unhealthy condition. At the same time, he has admitted that he removed to-day a bridge of the old type around which there was considerable hypertrophy and a diseased condition. All of you have seen a great many cases in which there was a diseased or unsanitary condition, existing on account of the old so-called self-cleansing form of construction. These are matters of construction for each of us to work out individually and to handle them as intelligently as we can in practice. The question resolves itself to the kind of technic and practice that best suits one's own individual judgment and ability, so that I merely say this much in regard to the saddle again. Use it carefully. Do not go into it in a slipshod way and use it indiscriminately, but try and get a proper knowledge of the cases in which these saddles are to be used and use them intelligently, and I assure you with all earnestness you will not be disappointed. —Dr. Roach in *Dental Review.*

*Common Colds and Vaccine Therapy.* Fisher is convinced that colds, so-called, are unquestionably due to infection by micro-organisms and therefore are contagious. They can largely be prevented by reasonable isolation of every case, and preventive inoculation. They can be aborted or their course shortened by vaccine treatment; in fact, the treatment of acute and chronic inflammation of the respiratory tract by vaccines is specific. —*Journal A. M. A.*, June 21, 1913.

*Life Conservation.* The paradox has been well put that the most precious thing is the cheapest—in money; while the most useless thing is the dearest—in dollars and cents. The cost of killing a man in modern warfare averages $15,000; in the Boer War it was $40,000. The six great European powers are grinding two billions of dollars out of their respective peoples for military preparation—not for war, it is said, but to prevent war. Meanwhile millions of young men who should be engaged in industrial pursuits are maintained in utterly unprofitable idleness, and are quartered, it is declared, in inadequate and sometimes tuberculosis-ridden barracks. On the other hand, Colonel Gorgas and his associates have converted the formerly pestilent and deadly Isthmus of Panama into one of the healthiest regions on earth, at a cost of $2.43 for each life saved. Many hundreds of men, women and children in our

South have been cured of the hookworm disease at an average cost of 77 cents for each sufferer. It is indeed true that the most precious thing in nature is human life (and human health by which life is enjoyed and lengthened), and as we have seen, it is about the cheapest; while the most useless and foolish thing is human warfare, which is the most costly.

Irrationality akin to that evinced in Europe—though not so flagrant—has been shown by our House Committee on Indian Affairs in refusing the request of the Secretary of the Interior for adequate funds with which to do hospital sanitation and medical work among the "nation's wards." The appropriation has been $90,000; but investigation revealed conditions much worse than had been supposed. The death rate from pulmonary tuberculosis in the federal registration area is 11; among the Indians it is 32, while the death-rate of the latter from all causes is 30, or more than double that in the registration area. On ascertaining these facts Secretary Fisher in his annual report recommended that the appropriation for prevention and treatment of disease be increased to $400,000; but the members of our billion-dollar Congress (which had just voted millions to be added to those already given in pensions) balked at such expenditure in behalf of a people for whose welfare our government has very largely assumed the responsibility and whose diseases are constantly endangering their white neighbors.

The conservation of health is perhaps the most important idea which the twentieth century has thus far evolved. Irving Fisher computed that the span of life in the United States could be increased fifteen years if all the hygienic reforms now known were put into effect, and outlined a plan for better health protection by federal, state and municipal governments, with the co-operation of many agencies and movements now benignantly active for the betterment of sanitary and hygienic conditions among our people. The life insurance companies have taken admirable part, and have conceived a policy of enlightened selfishness, based on the fact that the longer the policy holder lives, the more premiums he will pay. Mr. Cox, of New York, the counsel of the Association of Life Insurance Presidents, states that out of a total of nearly thirty million policies in force in American Companies at the end of 1910, the companies in this association carry about 77 percent. (or over twenty-three million), and of the latter, 97 percent. are in companies now engaged in individual work for health improvement. Five companies within this association (having policies aggregatin $22,000,000) make special efforts to stimulate their policy holders to activities in personal and public hygiene—mostly by articles in company periodicals distributed to policy holders and by other effective literature. One company has done this for many years. Another co-operates besides with existing anti-tuberculosis and like agencies, for health improvement; this company is experimenting in many cities with visiting nurses to sick policy holders. Another company has established a department of conservation which is ambitious, among other things, to aid public health authorities in the fight against preventable disease. This is a tremendous activity indeed, and with plenty of material to work on.; for actuaries have estimated that the economic value of lives lost needlessly each year in the United States alone is $1,500,000,000.—*Journal A. M. A.*, June 28, 1913.

*The Specialist..* The man who can become the greatest specialist in any one branch of medicine or dentistry is the man who has become great in general practice, but whose ambitions and tendencies lead him to condense his energies to one specialty. With this experience he is conservative in diagnosis, bold and fearless in his field of operation, and usually brings about the most satisfactory results.—W. D. Tracy, in *Journal Allied Societies.*

*Autogenous Vaccines in Chronic Joint Affections.* Rheumatoid arthritis is, Hughes says, a misleading term. He would prefer to call the condition "metastatic arthritis" —that is, metastatic to some infective focus in the body. Pathologically rheumatoid arthritis presents features of a chronic infection, the synovial membrane is pinker than normal, it is soft and succulent, and often villous processes project from it. The articular ends of the bones beneath the articular cartilage show rarefaction, while microscopically there is present some extent of round-celled infiltration in the synovial membrane. Although the synovial membrane is thickened, it does not show a tendency to ulcerate or undergo destructive changes as in the case of tubercle, and possibly syphilis. The three main points in the treatment of this disease are: 1. To find the infecting organisms. 2. Having found them, to raise the opsonic index of the individual against these organisms; this is most quickly accomplished by the use of autogenous vaccines. 3. Having rendered the patient artificially immune, to remove where possible the primary focus and apply local treatment to the affected joints. The commonest foci of infection in Hughe's cases have been the teeth, the nose and nasopharynx, chronic otorrhea, the lungs, the intestinal tract and leucorrhea.—*Journal A. M. A.*, July 12, 1913.

*Professional Advice.* Never be afraid to make a fee for professional advice. The dentist who neglects to instruct his patient in the proper, every-day care of the teeth fails in his capacity as dentist to the patient. Most beautiful and accurate restorations have failed in an alarmingly short time because of ignorance on the part of the patient in keeping the mouth in a hygienic condition. It is far easier to prevent dental troubles than to cope with the disasters following neglect.—W. D. Tracy, in *Journal Allied Societies.*

*Erosion of the Teeth and General Pathology.* Coustaing and Filderman give detailed histories of sixty-four cases of erosion of the teeth, calling attention to the inevitable coincidence of other allied stigmata of degeneration. The list includes malformations of the hands, teeth and ears, prognathism, scoliosis, delayed and disordered menstruation or other abnormalities indicating a more or less profound disturbance in the development of the individual which is to be attributed to hereditary influences. They have never found a case of erosion in an otherwise perfectly normal body, and conclude that it can not be due to any transitory infection, but only to profound hereditary causes. Every child with erosion of the teeth should be submitted to a thorough physical examination.—*Journal A. M. A.*, July 12, 1913. From *Review de Medicine*, Paris.

*Rubber Cements.*

I

| | |
|---|---|
| Guttapercha | 20 grammes |
| Caoutchouc | 40 grammes |
| Isinglass | 10 grammes |
| Carbon disulphide | 160 grammes |

II

| | |
|---|---|
| Caoutchouc | 4 grammes |
| Rosin | 8 grammes |
| Japan wax | 6 grammes |
| Benzin | 16 grammes |

—*Dental Scrap Book.*

*A Perfect Denture.* A few days ago a young man came to me to have his teeth examined. He said it was his first vist to a dentist. I was surprised to find a complete denture of pearly white teeth. In a practice of thirty years I have never seen so fine a set of teeth. The gums were perfect and there were no stains or calcular deposits. I asked him if he had been accustomed to eat sweets as a young man. He said I am twenty years of age and was raised in the back country of Kansas. My diet was principally corn bread and vegetables and my exercise hard work. I tasted ice cream the first time when I was sixteen years old. I can't remember ever tasting candy as a boy. His teeth looked more like a solid rare quality of quartz. He is studying medicine and if he makes as good a doctor as his teeth warrant, he will do.—C. R. Sabin, in *Pacific Gazette.*

